# Supplementary figures and images for: A Rare MSH2 Variant as a Candidate Marker for Lynch Syndrome II Screening in Tunisia: A Case of Diffuse Gastric Carcinoma
Source: Genes (Basel). 2022 Jul 28;13(8):1355. doi: 10.3390/genes13081355 (PMC9407052; doi:10.3390/genes13081355)

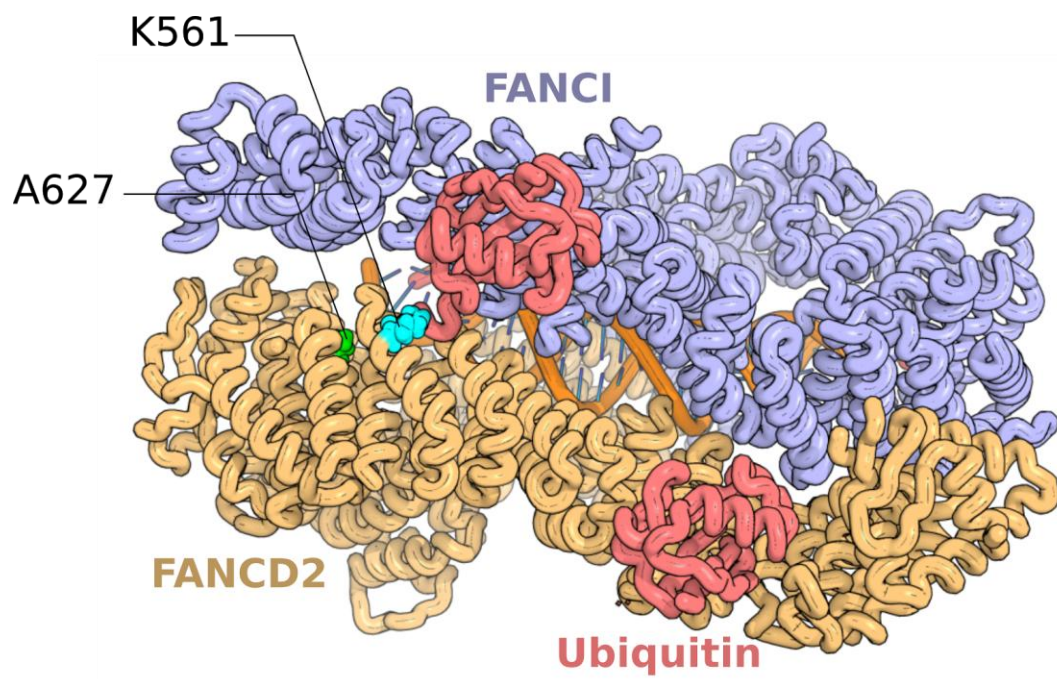

Supplement: Supplementary file 1 [file genes-13-01355-s001.zip › Figure S1.pdf]
